# Achieving dynamic behaviour and thermal expansion in the organic solid state *via* co-crystallization†
†Electronic supplementary information (ESI) available: Experimental details, single-crystal X-ray data and structures, DSC thermograms, and PASCal thermal expansion results. CCDC 1026670–1026679 and 1400641. For ESI and crystallographic data in CIF or other electronic format see DOI: 10.1039/c5sc00988j



**DOI:** 10.1039/c5sc00988j

**Published:** 2015-05-26

**Authors:** Kristin M. Hutchins, Ryan H. Groeneman, Eric W. Reinheimer, Dale C. Swenson, Leonard R. MacGillivray

**Affiliations:** a Department of Chemistry , University of Iowa , Iowa City , Iowa 52242-1294 , USA . Email: len-macgillivray@uiowa.edu ; Fax: +1-319-335-1270; b Department of Biological Sciences , Webster University , St. Louis , MO 63119 , USA; c Department of Chemistry and Biochemistry , W. M. Keck Foundation Center for Molecular Structure , California State University San Marcos , San Marcos , CA 92096 , USA

## Abstract

Thermal expansion involves a response of a material to an external stimulus that typically involves an increase in a crystallographic axis (positive thermal expansion (PTE)), although shrinking with applied heat (negative thermal expansion (NTE)) is known in rarer cases. Here, we demonstrate a means to achieve dynamic molecular motion and thermal expansions in organic solids *via* co-crystallizations. One co-crystal component is known to exhibit dynamic behaviour in the solid state while the second, when varied systematically, affords co-crystals with linear thermal expansion coefficients that range from colossal to nearly zero. Two co-crystals exhibit rare NTE. We expect the approach to guide the design of molecular solids that enable predesigned motion related to thermal expansion processes.

## Abstract 2

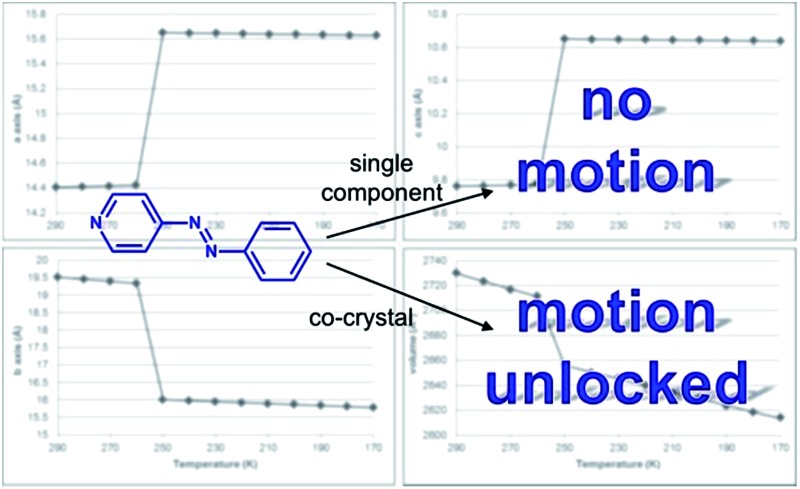
Molecular motion of an azo functional group is ‘unlocked’ *via* co-crystallizations.

Systematic modification of solids can aid in establishing reliable structure/property relationships,^1^ although the design of molecular materials that display predictable thermal expansion (TE) properties represents an ongoing and major challenge. Solids that exhibit controllable thermal expansion properties would be desirable in the design of thermomechanical actuators, sensors, and composites.^2^ The incorporation of an organic functional group known to undergo molecular movement when exposed to an external stimulus and when incorporated into a co-crystal can, in principle, be an attractive means to achieve movement and/or expansion properties in organic solid-state materials. In this context, the azo group is known to undergo pedal motion in the solid state,^3^ but, to our knowledge, has remained unexplored as a means to control thermal expansion properties of organic solids. Co-crystallization, the process of combining at least two neutral chemical species that are solids at ambient conditions,^4^ offers opportunities to modify thermal properties of solids^5^ based on azo groups wherein pedal motion may be absent in the single component.^3c,6^ Edge-to-face^7^ and face-to-face^8^ stackings of aromatics, and transitions between them,^9^ can also significantly impact expansion properties of organic solids, particularly in the context of organic electronics (*e.g.* acenes).^10^ We demonstrate here an application of these ideas in a series of organic co-crystals that exhibit different degrees of dynamic molecular motion and TE coefficients that span a wide range (Fig. 1).

**Fig. 1:**
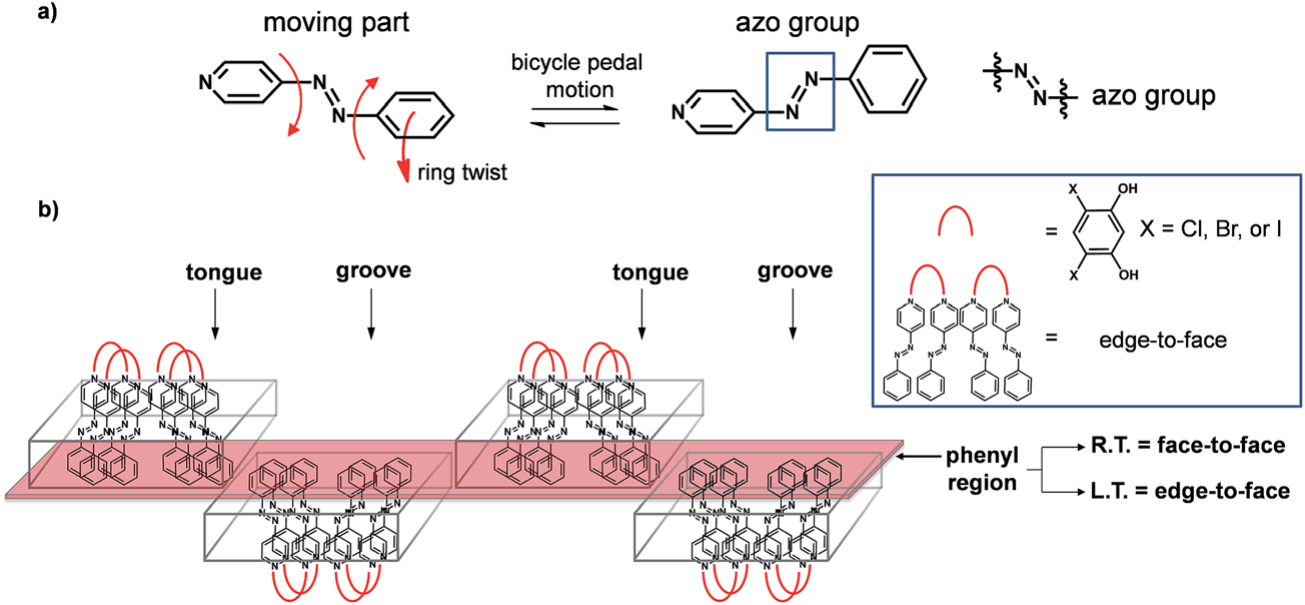
Dynamic motion of azo groups in a series of co-crystals: (a) reversible pedal motion with ring twists and (b) tongue-and-groove structure highlighting twisting that occurs in a phenyl region (red).

Specifically, we employ a ditopic co-crystal former (CCF) to furnish crystalline environments based on layering of hydrogen-bonded supramolecular assemblies that support molecular pedal motion of the azo group. Collective motion involving the azo groups leads to facile and widespread interconversion of face-to-face and edge-to-face stackings of aromatics that is facilitated by pedal motions in close-packed environments. We are unaware of a series of organic solids in which thermal properties have been readily modified by varying the second component of a co-crystal.

The azo group is present in 4-phenylazopyridine (**4PAzP**) (Fig. 1a).^11^ Single crystals of **4PAzP** were grown from ethanol and X-ray analysis revealed the molecule to crystallize in the chiral orthorhombic space group *P*2_1_2_1_2_1_. The extended packing of **4PAzP** is dominated by C–H···N interactions that propagate in a zigzag pattern along the *a*-axis and edge-to-face packing dominates along the crystallographic *b*-axis (Fig. 2). At 290 K, the azo core is disordered [site occupancies 0.87(1) and 0.13(1)], and the site occupancies remain unchanged when the crystal is cooled to 170 K. All crystallographic axes decreased slightly (PTE) when the crystal was cooled, a common behaviour in molecular solids. There was no evidence of a phase transformation during the cooling cycle, while DSC confirmed no phase transformation upon cooling (see ESI, Fig. S5†). PASCal^12^ was used to calculate the principal axes and linear TE coefficients in **4PAzP**, and revealed coefficients of TE as nearly zero. The azobenzene **4PAzP** packs in a herringbone motif in the crystallographic *ab*-plane.

**Fig. 2:**
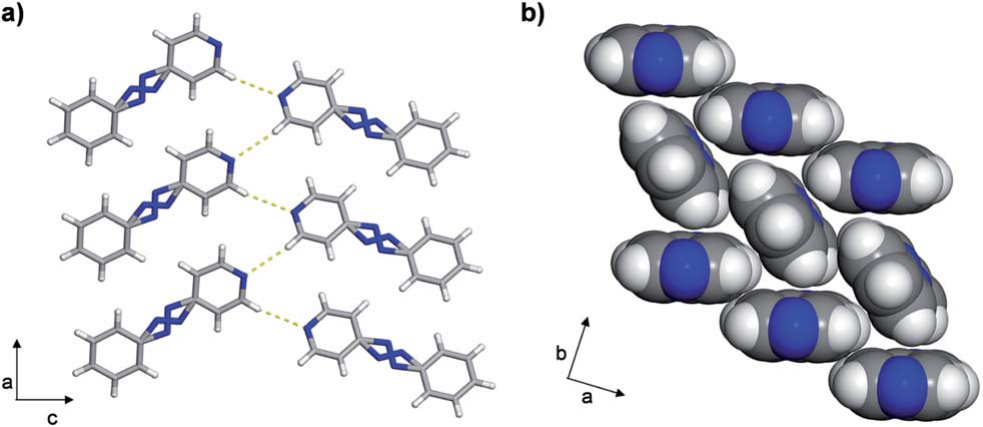
Crystal structures of **4PAzP** at 290 K: (a) C–H···N interactions and (b) herringbone packing.

To achieve dynamic motion in the solid state, we turned to a co-crystallization strategy^13^ involving **4PAzP**. Thus, co-crystallization of **4PAzP** with the ditopic receptor 4,6-dichlororesorcinol (**4,6-diCl res**) (Fig. 1b) in a 2 : 1 ratio in ethanol yielded single crystals overnight suitable for X-ray diffraction.

Single-crystal analysis revealed the three-component supramolecular assembly 2(**4PAzP**)·(**4,6-diCl res**) **1** that crystallizes in the monoclinic space group *P*2_1_/*c* and is sustained by two O–H···N hydrogen bonds (O···N (Å): 2.734(1), 2.747(1)). One azo moiety is disordered (**azoA**) over two sites [site occupancies: 0.61(1) and 0.39(1)] while the second azo group is ordered (**azoB**) (Fig. 3a). The major conformer in **azoA** lies parallel with **azoB**, and both **4PAzP** molecules lie nearly planar. The pyridine and benzene rings deviate from coplanarity by 12.6° and 18.2° for the disordered and ordered molecules, respectively. At room temperature (290 K), the aromatic rings within the hydrogen-bonded assembly in **1** are nearly co-planar, with the two azobenzenes interacting through face-to-face π–π forces. The pyridine rings lie nearly orthogonal to **4,6-diCl res** at 84.7° and 87.6° for the disordered and ordered groups, respectively.^13*a*^

**Fig. 3:**
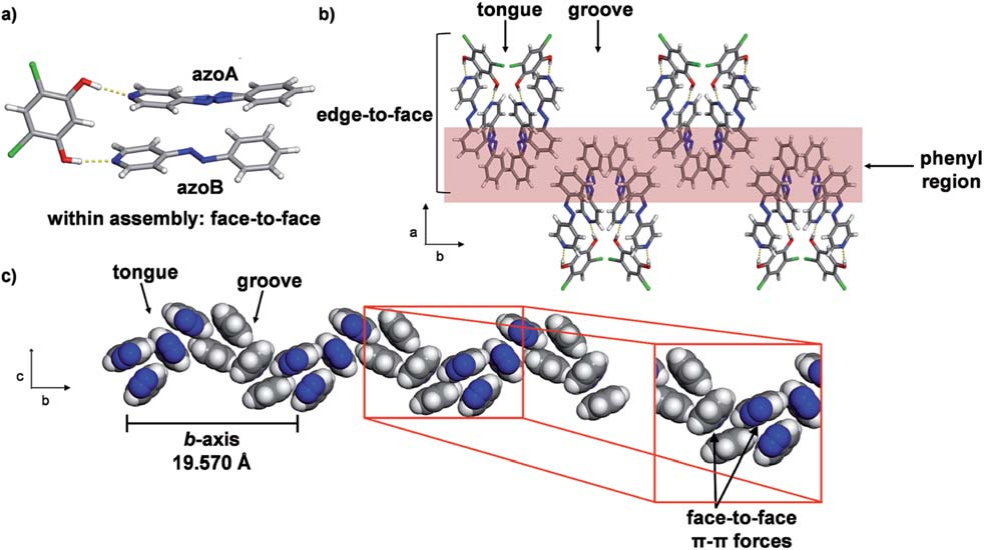
Co-crystal **1** at 290 K: (a) three-component assembly, (b) edge-to-face forces that define tongue-and-groove, and (c) end-on view of face-to-face forces within assemblies (blue = azo group).

The three-component assemblies alternate ‘head-up, tail-up’ along the *b*-axis (where: head = res/pyridine and tail = phenyl) to lie interdigitated in a tongue-and-groove type structure.^14^ The **4PAzP** azobenzene molecules are organized tail-to-tail within the *bc*-plane to define a 2D sheet based on phenyl–phenyl interactions in the two dimensions (Fig. 3b). The 2D sheet extends along the *b*-axis through the face-to-face π-interactions in each assembly and the edge-to-face phenyl–phenyl π-interactions between assemblies (Fig. 3c). The tongue and groove are, thus, effectively joined by the edge-to-face forces.

Importantly, when a variable temperature X-ray diffraction study was performed in the temperature range 290–170 K, molecular pedal motion involving **4PAzP** occurred in **1**. More specifically, a single-crystal-to-single-crystal (SCSC) isosymmetric phase transition^15^ was realized between 260 and 250 K. While the crystal system and space group were retained, there were dramatic changes in all three crystallographic axes. The *a*- and *c*-axes expanded, while the *b*-axis contracted (see ESI, Table S1†). The axes, thus, experienced negative (NTE) (*a*- and *c*-axes) and positive (PTE) (*b*-axis) thermal expansions. While the expansions in the *a*- and *c*-axes were substantial (1.2 and 0.9 Å respectively), corresponding to increases of 8.7 and 9.2%, respectively, the contraction of the *b*-axis (3.58 Å) was colossal, corresponding to an 18.3% decrease from the original size. Despite the large changes in the cell axes, however, the volume of the single crystal decreased by only 82 Å^3^ or approximately 3%.^2a,16^

The X-ray structure at 250 K‡
‡Crystal experiences phase transition at 260 K. A description of the crystal structure after the phase transition at 250 K is provided. revealed a three-component assembly sustained by two O–H···N hydrogen bonds (O···N (Å): 2.704(1), 2.715(1)) (Fig. 4a). In the solid, the formerly disordered azo group (**azoA**) of **4PAzP** is fully occupied while the pyridine and benzene rings for **azoA** and **azoB** underwent an approximate 30° twist relative to one another. Additionally, while the pyridine ring attached to **azoB** underwent a moderate 14° twist with respect to **4,6-diCl res**, the pyridine attached to **azoA** underwent a dramatic 64° twist. The large twist was accompanied by both azo units undergoing pedal motions within the solid, with the azo groups assuming a criss-crossed geometry within the hydrogen-bonded structure. The twisting and pedal motions resulted in the formation of two C–H···N hydrogen bonds involving a *meta*- and *ortho*-hydrogen atom of a pyridine and phenyl ring with the two N atoms of a N=N unit (C···N (Å): 3.780, 3.900) of a hydrogen-bonded structure. The motion exhibited by the azobenzene components of an assembly effectively resulted in the face-to-face stacking dramatically converting to an edge-to-face π–π structure (Fig. 4b).

**Fig. 4:**
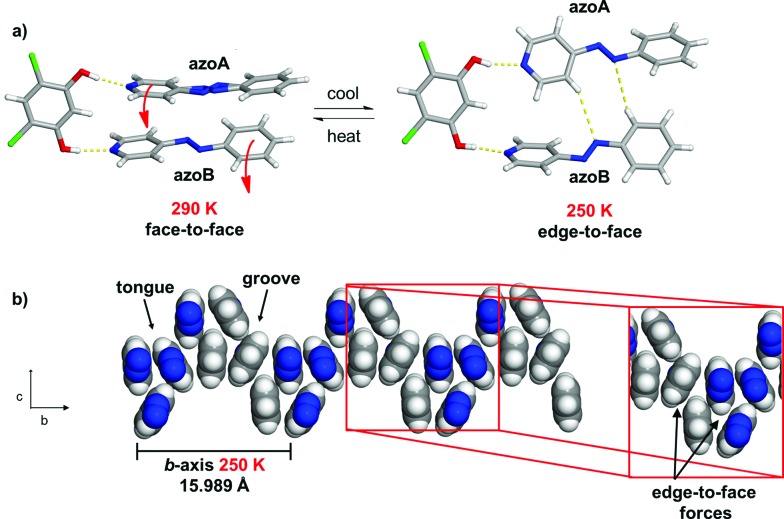
Co-crystal **1** at 250 K: (a) conversion of face-to-face to edge-to-face stacking of a hydrogen-bonded assembly and (b) π-interactions of azobenzenes with edge-to-face forces within an assembly highlighted (blue = azo group).

The pedal motion and twisting of the rings within the hydrogen-bonded assembly account for the very large contraction of the *b*-axis and expansions of the *a*- and *c*-axes in **1**. The interconversion of the rings within the three-component assembly is propagated along the *b*- and *c*-axes such that the phenyl–phenyl interactions that define the 2D sheet effectively interconvert from an edge-to-face to face-to-face geometry within a groove and between the tongue-and-groove structures (Fig. 5a). Within a groove, neighbouring edge-to-face forces convert face-to-face and result in the formation of two C–H···N hydrogen bonds between **azoA** molecules of neighbouring assemblies (C···N (Å): 3.719, 3.722) (Fig. 5b and c). The generation of the inter-assembly hydrogen bonds produces a step-like structure that extends within the *bc*-plane. Thus, upon cooling, the **4PAzP** aromatic rings undergo a ‘twist-and-slide’ *via* the C–H···N forces.^17^ The relative weakness of the C–H···N forces presumably supports the interactions to be reversed upon warming to room temperature. In essence, the ditopic **4,6-diCl res** serves as a spatially-fixed axle that supports cooperative rotation of the **4PAzP** molecules throughout the lattice.^18^ The main contributor to the expansion along the *a*-axis results from changes in inter-Cl distances between **4,6-diCl res** molecules. From 290 to 250 K, the distance between the Cl atoms increases from 3.74 to 4.90 Å (*i.e.* 1.16 Å), which allows for large twisting of the **4PAzP** molecules. We are unaware of an example wherein such widespread interconversion of edge-to-face and face-to-face π–π stackings of aromatics accompanies thermal expansions in a molecular solid.

**Fig. 5:**
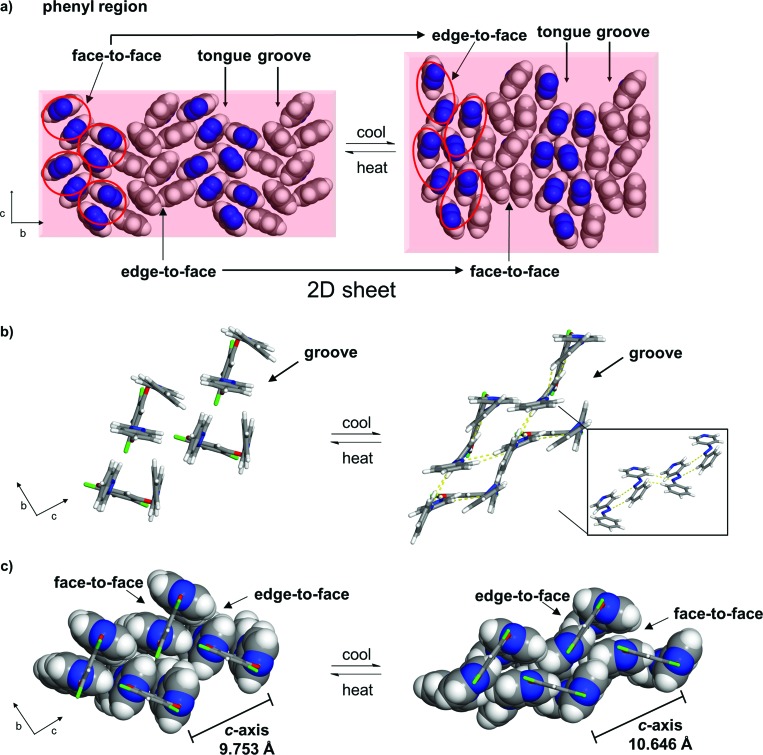
Interconversion of π–π stacking in **1** (290 to 250 K): (a) 2D sheet (rotation of phenyl region by 60° relative to Fig. 1; circles highlight azo groups of an assembly), (b) C–H···N interactions (yellow dotted lines, inset shows step-like structure) and (c) interconversions between edge-to-face and face-to-face π–π packings within a groove (resorcinol axle shown in wireframe).

The NTE and PTE involved in the interconversion of the edge-to-face and face-to-face stackings is remarkably large for an organic solid. PASCal^12^ was used to calculate the principal axes and linear TE coefficients for **1** in two temperature ranges 290–260 and 250–170 K using unit cell parameters collected from a crystal of **1** at 10 K increments (Fig. 6). From 290–260 K, the linear TE coefficients for **1** are –116, 29, and 316 MK^–1^ for *X*_1_, *X*_2_, and *X*_3_, respectively, with *X*_1_, *X*_2_, and *X*_3_ approximately along [203], [10–1], and [010] crystallographic axes. The volumetric TE coefficient over the temperature range is 229 MK^–1^. The negative component of TE is comparable to the value reported for a solvent inclusion complex, which is also based on multiple components.^19^ The coefficients for the positive linear TE *X*_3_, which corresponds to the crystallographic *b*-axis, and the volumetric TE are comparable to the largest reported TE coefficients for organic-based compounds.^1e,2a,5,19,20^ We note that the site occupancies of **azoA** change on the order of 5% in the temperature range, while the pyridine and benzene rings undergo twisting toward planarity. The phenyl rings also undergo twisting both within a groove and between the tongue-and groove structure. Collective dynamic molecular motion was, thus, realized among the hydrogen-bonded components, which contribute to the positive and negative TE between 290 and 260 K. The abrupt change in cell axes between 260–250 K corresponds to a phase transition, and following the transition, **1** showed PTE. From 250–170 K, the linear TE coefficients for *X*_1_ and *X*_2_ are nearly zero, 0.5 and 28 MK^–1^, respectively. However, *X*_3_, which is only dominated by changes in the crystallographic *b*-axis, is 174 MK^–1^. The volumetric TE coefficient is 203 MK^–1^, and both of these PTE values are also quite large for organic-based compounds.^1e,2a,5,20a,21^ We emphasize that the dynamic motion of the azo moiety and accompanying colossal negative and positive thermal expansions are only realized *via* the co-crystallization experiments.

**Fig. 6:**
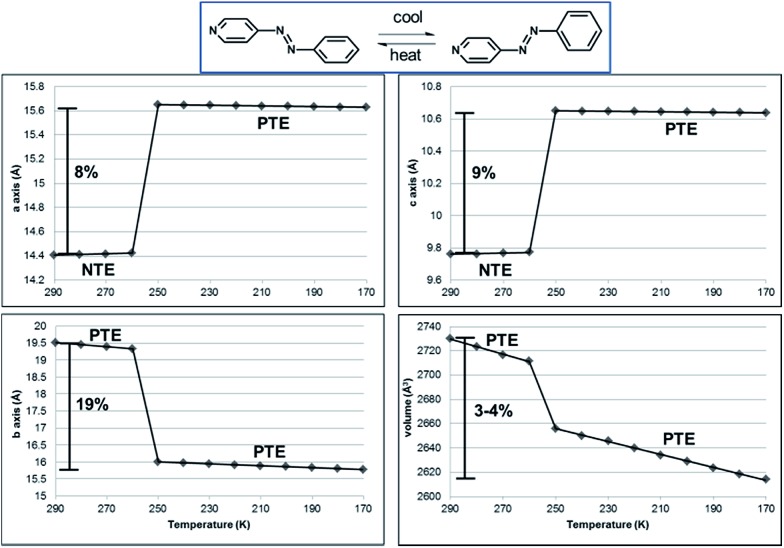
Unit-cell parameters as a function of temperature for **1**. NTE along the *a*- and *c*-axes and PTE along the *b*-axis and unit-cell volume, as highlighted. Linear fits of the data provided.

When differential scanning calorimetry (DSC) was performed on a powdered sample of **1** starting at room temperature, an exothermic phase transformation in line with the X-ray data was observed at 250 K. Upon warming to room temperature from 215 K, an endothermic phase transformation was observed at 273 K. The solid, thus, exhibited a thermal hysteresis of 23 K, likely indicating a first order structural phase transition.^22^ The cycle could be repeated for at least 15 consecutive runs (see ESI, Fig. S6†).

Interconverting face-to-face and edge-to-face stacking, as well as dynamic motion involving **4PAzP**, were also realized in the closely-related co-crystal of **2** based on 4,6-dibromoresorcinol (**4,6-diBr res**). Co-crystallization of **4PAzP** and **4,6-diBr res** in a 2 : 1 ratio in ethanol yielded single crystals suitable for X-ray analysis.

Single-crystal analysis at 290 K revealed a three-component supramolecular assembly with the formula 2(**4PAzP**)·(**4,6-diBr res**) **2**. The components crystallized in the monoclinic space group *P*2_1_/*c* to form a solid sustained by O–H···N hydrogen bonds (O···N (Å): 2.725(1), 2.731(1)) (see ESI, Fig. S2†) that is isostructural to **1**. Similar to **1**, the asymmetric unit of **2** contains one azo molecule that is disordered (**azoC**) over two sites [site occupancies: 0.56(1) and 0.44(1)], as well as an ordered azo unit (**azoD**). The major conformer in **azoC** lies criss-crossed to **azoD**, in contrast to the azo groups of **1**.

A variable temperature X-ray diffraction study performed on **2** (290–170 K) revealed molecular pedal motion to also occur. Upon cooling, a SCSC isosymmetric phase transition^15^ was realized near 250 K. Following the SCSC transformation, two salient changes were relevant to the thermal behaviour. Specifically, the *b*-axis displayed a much less dramatic PTE and the *a*-axis underwent PTE during the phase transition as opposed to NTE for **1** (see ESI, Table S2, Fig. S1†). The *c*-axis underwent NTE as in **1**, and the relative changes in all three axes were substantial. The contractions in the *a*- and *b*-axes (1.0 and 0.7 Å respectively), correspond to decreases of 6.7 and 3.2%, respectively, while the expansion of the *c*-axis (0.7 Å), corresponds to an 8.2% increase from the original size. The volume of the single crystal decreased by 112 Å^3^, or approximately 4%. The differences in TE properties between **1** and **2** can be attributed to the presence of Br···Br interactions (3.64 Å),^23^ as compared to the absence of halogen···halogen forces in **1**, that lie approximately along the crystallographic *a*-axis. The Br···Br forces remain intact upon cooling, which likely does not provide sufficient room for large twisting of the aromatic rings (Fig. 7) (see ESI, Fig. S3†). Less dramatic twisting was also realized by the aromatic rings in **2** compared to **1**, while both azo units did undergo pedal motion upon cooling, albeit having switched from crossed to parallel. The stacked rings within the assembly of **2** interconvert from face-to-face to edge-to-face so as to form only one C–H···N hydrogen bond between the *ortho*-hydrogen of a phenyl group and a N atom of a N=N unit (C···N (Å): 3.913). Within a groove, the edge-to-face π-interactions switch to face-to-face and one C–H···N hydrogen bond between **azoC** molecules in neighbouring assemblies (C···N: 3.252 Å) has formed. The contraction along the *a*-axis is realized by twisting of the pyridine and benzene rings towards planarity with neighbouring **res** molecules.

**Fig. 7:**
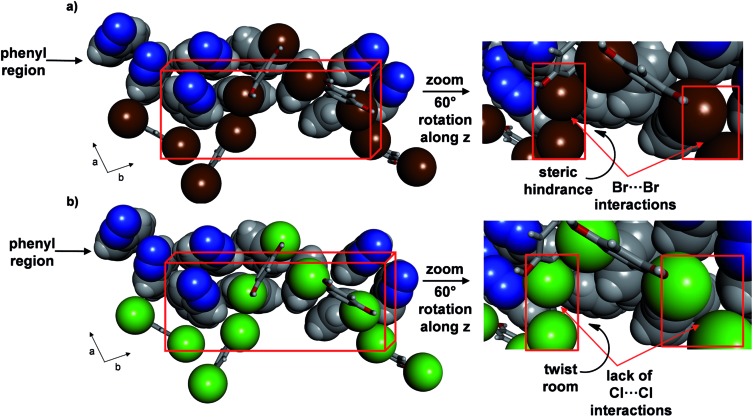
Crystal structures at 290 K highlighting role of halogen interactions in phenyl region of: (a) **2** and (b) **1** (right provides insets).

Linear TE coefficients for **2** were calculated^12^ in the temperature ranges of 290–260 and 250–170 K using unit cell parameters collected from a single crystal of **2** at 10 K increments. From 290–260 K, the linear TE coefficients for **2** are –51, 58, and 193 MK^–1^ for *X*_1_, *X*_2_, and *X*_3_, respectively, with *X*_1_, *X*_2_, and *X*_3_ approximately along [100], [010], and [001] crystallographic axes. The volumetric TE coefficient over the range of 290–260 K is 201 MK^–1^. In the 290–260 K range, the PTE coefficient corresponding to the crystallographic *b*-axis is an order of magnitude smaller than in **1**, as supported by incomplete interconversion to face-to-face stacking along the *b*-axis. Less dynamic motion was also realized in **2** as compared to **1** in the temperature range. Specifically, while the site occupancies of **azoC** remained nearly intact, the pyridine and benzene rings underwent slight twisting both toward and away from planarity for the ordered and disordered species, respectively. As in **1**, the abrupt change in cell axes occurs during the phase transition in the temperature range of 260–250 K. In the range of 250–170 K, **2** showed PTE and the largest linear TE coefficient over the range is 125 MK^–1^, which is dominated by changes in the crystallographic *b*-axis. The volumetric TE coefficient is 249 MK^–1^, and the coefficients are appreciable for organic-based compounds.^2a,5,20^ Similar to **1**, DSC showed a reversible phase transformation with an exotherm at 245 K and an endotherm at 268 K (see ESI, Fig. S7†). The intrinsic dynamic behaviour of **4PAzP** and corresponding thermal expansion properties were, thus, further modified by changing the second component of the co-crystal.

Whereas **1** and **2** exhibit large thermal expansion coefficients, co-crystallization of **4PAzP** with 4,6-diiodoresorcinol (**4,6-diIodo res**) resulted in 2(**4PAzP**)·(**4,6-diIodo res**) **3** that undergoes minimal dynamic motion without a phase transition. Co-crystallization of **4PAzP** and **4,6-diIodo res** in a 2 : 1 ratio in ethanol yielded crystals suitable for X-ray analysis. A single-crystal analysis at 290 K revealed two unique three-component assemblies (Type I and Type II) sustained by O–H···N hydrogen bonds, wherein three of four azo groups are disordered (see ESI, Fig. S4†). The components crystallized in the triclinic space group *P*1; thus, **3** is not isostructural to **1** and **2**. The extended packing of **3** involves alternating parallel stacks of Type I and Type II assemblies along the *a*-axis and within the *bc*-plane. When the crystal was cooled from 290 to 190 K, all crystallographic axes slightly decreased (PTE). The X-ray structure at 190 K revealed no drastic molecular movements, although the site occupancies of the azo groups changed on the order of 10–15%, which is indicative of molecular pedal motion being retained.^6*a*^ A DSC analysis confirmed that a phase transformation did not occur upon cooling (see ESI, Fig. S8†). The coefficients of thermal expansion^12^ for **3** range from –13 to 135 MK^–1^. The use of the CCF **4,6-diIodo res** altered the assembly and packing such that twisting of aromatic rings and the formation of C–H···N interactions were not favoured.

## Conclusions

In conclusion, we have utilized a co-crystal strategy to achieve dynamic molecular motion and thermal expansion properties in a series of organic solids. An organic functional group that supports molecular motion in the solid state when co-crystallized with a series of CCFs has afforded solids that result in molecular pedal motion and widespread interconversion of face-to-face and edge-to-face aromatic stacks. The resulting thermal expansion coefficients range from colossal to nearly zero, with significant NTE realized in two solids. We are expanding the co-crystal approach with an aim to generate solids that exhibit dynamic molecular motion and affect thermal properties of organic solid-state materials.
