# Supplementary material for: Achieving dynamic behaviour and thermal expansion in the organic solid state via co-crystallization
Source: Chem Sci. 2015 May 26;6(8):4717–22. doi: 10.1039/c5sc00988j (PMC5500859; doi:10.1039/c5sc00988j)
Supplement: Supplementary file 1 [file SC-006-C5SC00988J-s001.pdf]

Electronic Supplementary Material (ESI) for Chemical Science.
This journal is © The Royal Society of Chemistry 2015

Achieving dynamic behavior and thermal expansion in the organic solid state *via* co-crystallization

Kristin M. Hutchins[a], Ryan H. Groeneman[b], Eric Reinheimer[c], Dale C. Swenson[a], and Leonard R. MacGillivray[a,*]

[a]Department of Chemistry, University of Iowa, Iowa City, IA 52242-1294, USA, [b]Department of Biological Sciences, Webster University, St. Louis, MO 63119, USA, [c]Department of Chemistry and Biochemistry, W.M. Keck Foundation Center for Molecular Structure, California State University San Marcos, San Marcos, CA 92096, USA

*Author email address: len-macgillivray@uiowa.edu

Supplementary Information:

1) Experimental information
2) Single crystal X-ray diffraction measurements
3) Unit cell parameters for co-crystal **1** and **2** at 10 K intervals
4) Single-crystal X-ray structures
5) Differential Scanning Calorimetry
6) Thermal expansion coefficients

1) Experimental Information

4PAzP was synthesized as reported.[1]

Preparation of crystals: Single crystals of **4PAzP** were grown from EtOH. Co-crystals were obtained by dissolving **4PAzP** (50 mg, 0.27 mmol) and the appropriate resorcinol (0.14 mmol) in EtOH. Slow evaporation over 1-2 days yielded single crystals suitable for X-ray diffraction.

2) Single crystal X-ray diffraction measurements

Single crystal XRD for the co-crystals was measured on a Nonius Kappa CCD single-crystal X-ray diffractometer using MoKα radiation (λ=0.71073 Å). Structure solution and refinement were accomplished using SHELXS and SHELXL, respectively.[2] All non-hydrogen atoms were refined anisotropically. Hydrogen atoms associated with carbon atoms were refined in geometrically constrained positions. Single crystal XRD data for the **4PAzP** data sets were measured on a Rigaku SCXMini diffractometer using MoKα radiation (λ=0.71073 Å). The structures were solved and refined using the same programs above within the Olex2[3] graphical user interface. All hydrogen and non-hydrogen atoms in **4PAzP** were treated in a manner analogous to those from the co-crystals.

Table S1. Crystallographic parameters for **4PAzP·4,6-diCl res** at variable temperatures.

compound name	**4PAzP·4,6-diCl res**	**4PAzP·4,6-diCl res**	**4PAzP·4,6-diCl res**	**4PAzP·4,6-diCl res**
chemical formula	$C_{28}H_{22}N_6O_2Cl_2$	$C_{28}H_{22}N_6O_2Cl_2$	$C_{28}H_{22}N_6O_2Cl_2$	$C_{28}H_{22}N_6O_2Cl_2$
formula mass	545.41	545.41	545.41	545.41
crystal system	Monoclinic	Monoclinic	Monoclinic	Monoclinic
space group	$P2_1/c$	$P2_1/c$	$P2_1/c$	$P2_1/c$
T/K	290(2)	270(2)	250(2)	170(2)
a/Å	14.400(6)	14.4187(14)	15.6347(16)	15.6181(16)
b/Å	19.510(8)	19.4064(19)	15.9890(16)	15.7722(16)
c/Å	9.757(4)	9.7692(9)	10.6460(11)	10.6301(11)
α/°	90.00	90.00	90.00	90.00
β/°	95.784(15)	95.552(5)	95.252(5)	95.137(5)
γ/°	90.00	90.00	90.00	90.00
V/Å^3	2727.2(19)	2720.7(5)	2650.1(5)	2608.0(5)
ρ_{calc} /g cm^{-3}	1.343	1.346	1.367	1.389
Z	4	4	4	4
radiation type	Mo $K_{\alpha1}$	Mo $K_{\alpha1}$	Mo $K_{\alpha1}$	Mo $K_{\alpha1}$
μ /mm^{-1}	0.275	0.276	0.283	0.287
no. of reflections measured	30069	29819	14606	22474
no. of independent reflections	4999	4944	4720	6180
no of reflection (I > 2σ(I))	2899	3071	3079	4701
R_{int}	0.0283	0.0261	0.0416	0.0395
R_1 (I > 2σ(I))	0.0438	0.0428	0.0508	0.0422
wR(F^2) (I > 2σ(I))	0.0966	0.0960	0.1023	0.0955
R_1 (all data)	0.0869	0.0811	0.1018	0.0651
wR(F^2) (all data)	0.1202	0.1196	0.1185	0.1070
CCDC deposition number	1026675	1026674	1026673	1026672

Table S2. Crystallographic parameters for **4PAzP·4,6-diBromo res** at variable temperatures.

compound name	**4PAzP·4,6-diBr res**	**4PAzP·4,6-diBr res**	**4PAzP·4,6-diBr res**
chemical formula	$C_{28}H_{22}N_6O_2Br_2$	$C_{28}H_{22}N_6O_2Br_2$	$C_{28}H_{22}N_6O_2Br_2$
formula mass	634.33	634.33	634.33
crystal system	Monoclinic	Monoclinic	Monoclinic
space group	$P2_1/c$	$P2_1/c$	$P2_1/c$
T/K	290(2)	270(2)	190(2)
a/Å	14.5095(15)	14.5204(15)	13.5422(13)
b/Å	20.268(2)	20.207(2)	19.6750(19)
c/Å	9.5541(10)	9.4966(9)	10.2805(10)
α/°	90.00	90.00	90.00
β/°	97.192(5)	97.176(5)	100.796(3)
γ/°	90.00	90.00	90.00
V/Å^3	2787.5(5)	2764.6(5)	2690.7(5)
ρ_{calc} /g cm^{-3}	1.511	1.524	1.5658
Z	4	4	4
radiation type	Mo $K_{\alpha1}$	Mo $K_{\alpha1}$	Mo $K_{\alpha1}$
μ /mm^{-1}	2.944	2.968	3.05
no. of reflections measured	14038	62803	33843
no. of independent reflections	4907	4217	4734
no of reflection (I > 2σ(I))	2711	2616	3920
R_{int}	0.0329	0.0489	0.0263
R_1 (I > 2σ(I))	0.0411	0.0551	0.0295
wR(F^2) (I > 2σ(I))	0.1169	0.0711	0.0893
R_1 (all data)	0.0982	0.1220	0.0430
wR(F^2) (all data)	0.1564	0.0920	0.1185
CCDC deposition number	1026671	1400641	1026670

Table S3. Crystallographic parameters for **4PAzP·4,6-diIodo res** and **4PAzP** at variable temperatures.

compound name	**4PAzP·4,6-diIodo res**	**4PAzP·4,6-diIodo res**	**4PAzP**	**4PAzP**
chemical formula	$C_{28}H_{22}N_6O_2I_2$	$C_{28}H_{22}N_6O_2I_2$	$C_{11}H_9N_3$	$C_{11}H_9N_3$
formula mass	728.31	728.31	183.21	183.21
crystal system	Triclinic	Triclinic	Orthorhombic	Orthorhombic
space group	*P-1*	*P-1*	$P2_12_12_1$	$P2_12_12_1$
T/K	290(2)	190(2)	290(2)	170(2)
a/Å	9.0480(9)	8.9820(9)	5.911(9)	5.9077(7)
b/Å	12.5933(13)	12.5271(13)	7.496(11)	7.4479(9)
c/Å	26.305(3)	26.146(3)	21.820(3)	21.752(3)
α/°	83.815(5)	84.500(5)	90.00	90.00
β/°	89.271(5)	89.331(5)	90.00	90.00
γ/°	75.005(5)	74.563(5)	90.00	90.00
V/Å^3	2878.0(5)	2822.4(5)	967.0(2)	957.1(2)
ρ_{calc} /g cm^{-3}	1.681	1.714	1.259	1.271
Z	4	4	4	4
radiation type	Mo $K_{\alpha 1}$	Mo $K_{\alpha 1}$	Mo $K_{\alpha 1}$	Mo $K_{\alpha 1}$
μ /mm^{-1}	2.221	2.264	0.079	0.080
no. of reflections measured	19210	38202	7882	7818
no. of independent reflections	10494	12943	2201	2193
no of reflection (I > 2σ(I))	6588	10557	1214	1339
R_{int}	0.0249	0.0194	0.0481	0.0425
R_1 (I > 2σ(I))	0.0557	0.0352	0.0559	0.0524
wR(F^2) (I > 2σ(I))	0.1585	0.0976	0.1245	0.1198
R_1 (all data)	0.0959	0.0487	0.1087	0.0959
wR(F^2) (all data)	0.2001	0.1233	0.1489	0.1411
CCDC deposition number	1026677	1026676	1026679	1026678

3) Unit cell parameters for co-crystal 1 and 2 at 10 K intervals

Table S4. Unit cell parameters from 290-170 K for co-crystal **1**. Unit cell determinations were performed on a single crystal at 10 K intervals.

Cell Dimensions	290 K	280 K	270 K	260 K	250 K
a (Å)	14.408(1)	14.412(1)	14.416(1)	14.422(1)	15.6506(9)
b (Å)	19.508(2)	19.451(1)	19.389(1)	19.325(1)	16.0014(9)
c (Å)	9.7623(7)	9.7625(7)	9.7661(7)	9.7741(6)	10.6508(6)
alpha (°)	90	90	90	90	90
beta (°)	95.774(2)	95.701(2)	95.618(2)	95.526(2)	95.244(2)
gamma (°)	90	90	90	90	90
volume ($Å^3$)	2730.0(6)	2723.2(5)	2716.7(5)	2711.5(5)	2656.1(4)

Cell Dimensions	240 K	230 K	220 K	210 K	200 K
a (Å)	15.6467(9)	15.6463(8)	15.6434(8)	15.6396(8)	15.6370(8)
b (Å)	15.9723(9)	15.9470(8)	15.9165(8)	15.8875(8)	15.8601(8)
c (Å)	10.6478(6)	10.6479(5)	10.6456(5)	10.6437(5)	10.6426(6)
alpha (°)	90	90	90	90	90
beta (°)	95.205(2)	95.199(2)	95.185(2)	95.173(2)	95.151(2)
gamma (°)	90	90	90	90	90
volume ($Å^3$)	2650.1(4)	2645.8(4)	2639.8(4)	2633.9(4)	2628.8(4)

Cell Dimensions	190 K	180 K	170 K
a (Å)	15.6344(8)	15.6320(8)	15.6307(8)
b (Å)	15.8331(8)	15.8071(8)	15.7833(9)
c (Å)	10.6414(5)	10.6392(5)	10.6384(6)
alpha (°)	90	90	90
beta (°)	95.139(2)	95.124(2)	95.110(2)
gamma (°)	90	90	90
volume ($Å^3$)	2623.6(4)	2618.4(4)	2614.1(4)

Table S5. Unit cell parameters from 290-170 K for co-crystal **2**. Unit cell determinations were performed on a single crystal at 10 K intervals.

**Cell Dimensions**	**290 K**	**280 K**	**270 K**	**260 K**	**250 K**
a (Å)	14.5154	14.5244	14.5323	14.5367	13.5515
b (Å)	20.2494	20.2399	20.2282	20.2139	19.7917
c (Å)	9.539	9.5207	9.5023	9.4844	10.3218
alpha (°)	90	90	90	90	90
beta (°)	97.178	97.175	97.173	97.1657	100.565
gamma (°)	90	90	90	90	90
volume (Å^3)	2781.8	2776.9	2771.5	2765.2	2721.5

**Cell Dimensions**	**240 K**	**230 K**	**220 K**	**210 K**	**200 K**
a (Å)	13.5473	13.5457	13.5437	13.5407	13.5339
b (Å)	19.7674	19.7476	19.7244	19.7002	19.6675
c (Å)	10.3131	10.305	10.2965	10.2875	10.2725
alpha (°)	90	90	90	90	90
beta (°)	100.6219	100.6688	100.7279	100.7645	100.81
gamma (°)	90	90	90	90	90
volume (Å^3)	2714.5	2708.9	2702.5	2695.9	2685.8

**Cell Dimensions**	**190 K**	**180 K**	**170 K**
a (Å)	13.5376	13.5292	13.5288
b (Å)	19.6519	19.6188	19.5985
c (Å)	10.27	10.2585	10.254
alpha (°)	90	90	90
beta (°)	100.847	100.88	100.903
gamma (°)	90	90	90
volume (Å^3)	2683.4	2673.9	2669.7

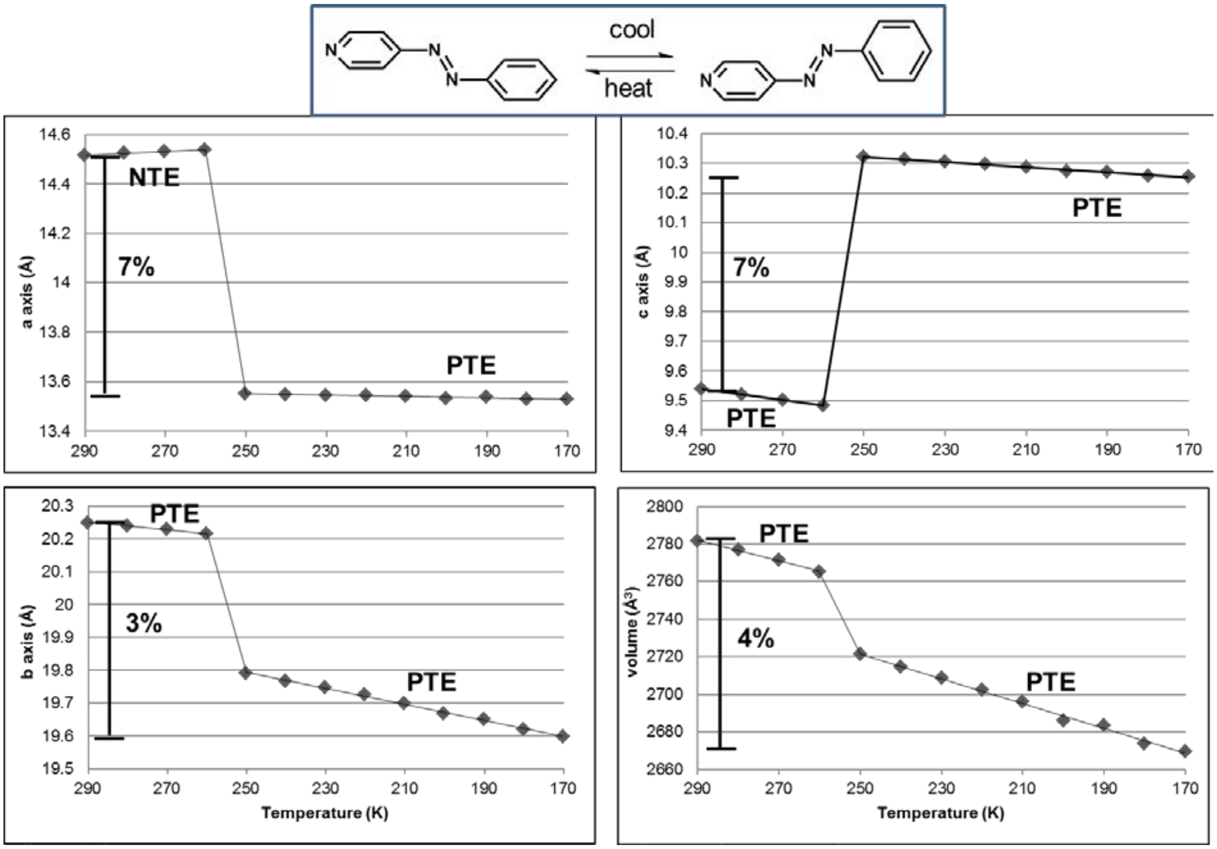

Fig. S1. Unit-cell parameters as a function of temperature for **2**. Linear fits of the data are provided.

4) Single-crystal X-ray structures

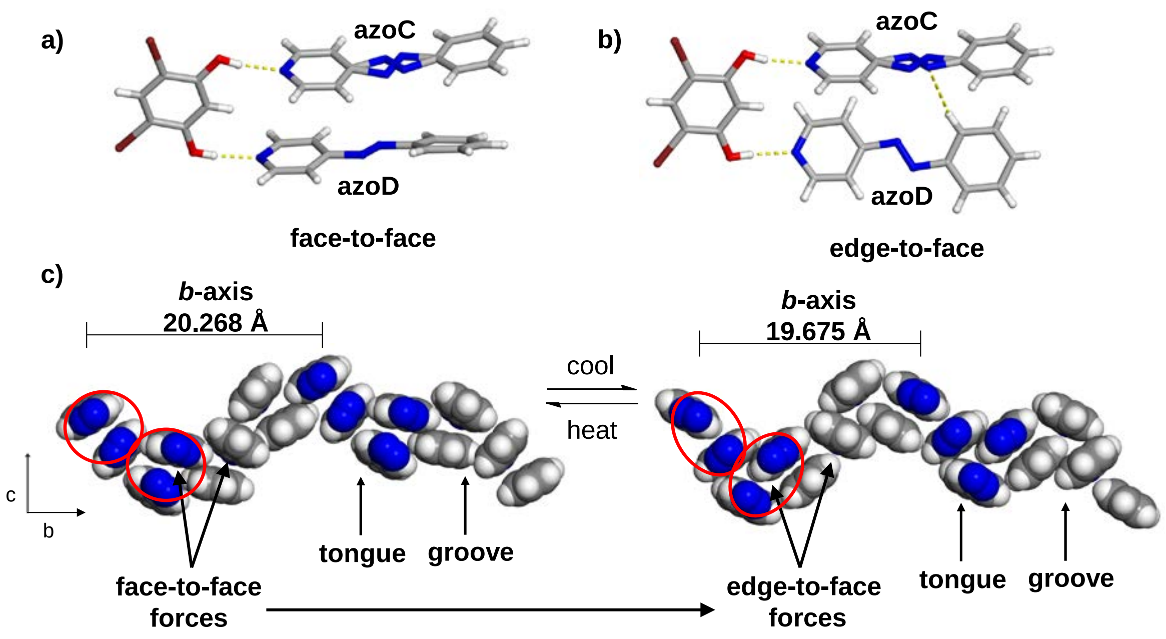

Fig. S2. Crystal structures of **2** (290 K to 190 K): (a) three-component assembly at 290 K, (b) three-component assembly at 190 K, and (c) interconversion of face-to-face and edge-to-face π-π interactions along the *b*-axis (circling corresponds to azo groups of an assembly).

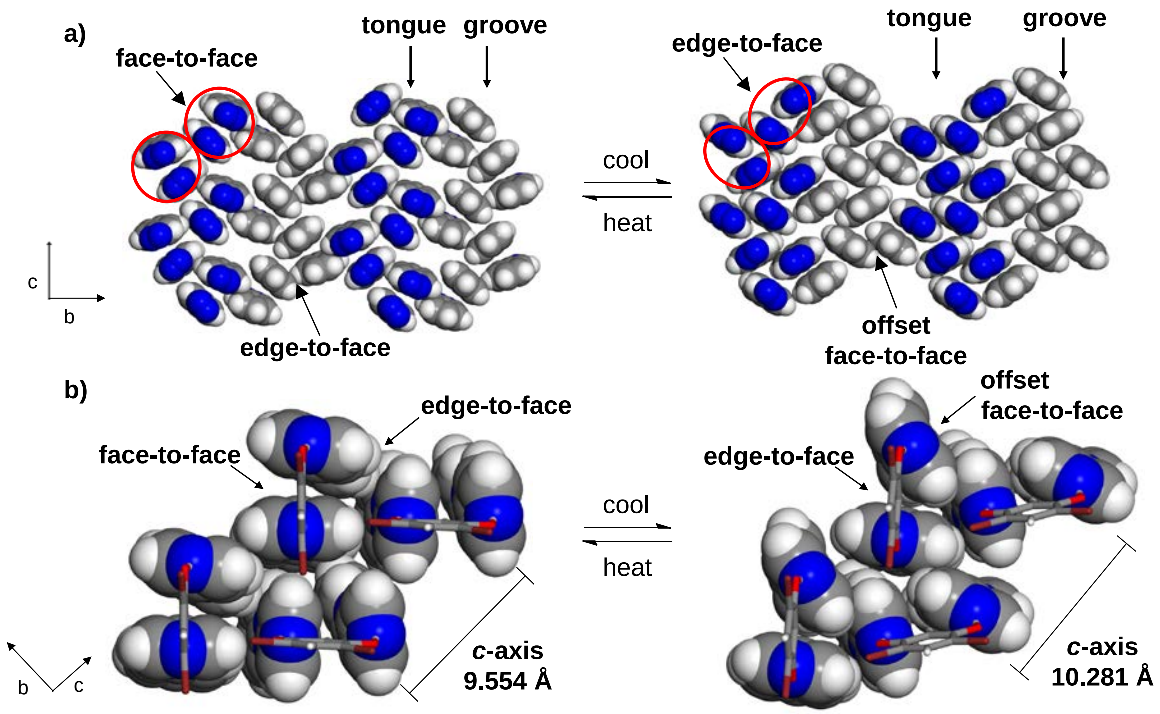

Fig. S3. Interconversion of π-π stacking in **2** (290 K to 190 K): (a) 2D sheet interactions (circling corresponds to azo groups of an assembly) and (b) interconversions between edge-to-face and face-to-face packings within a groove.

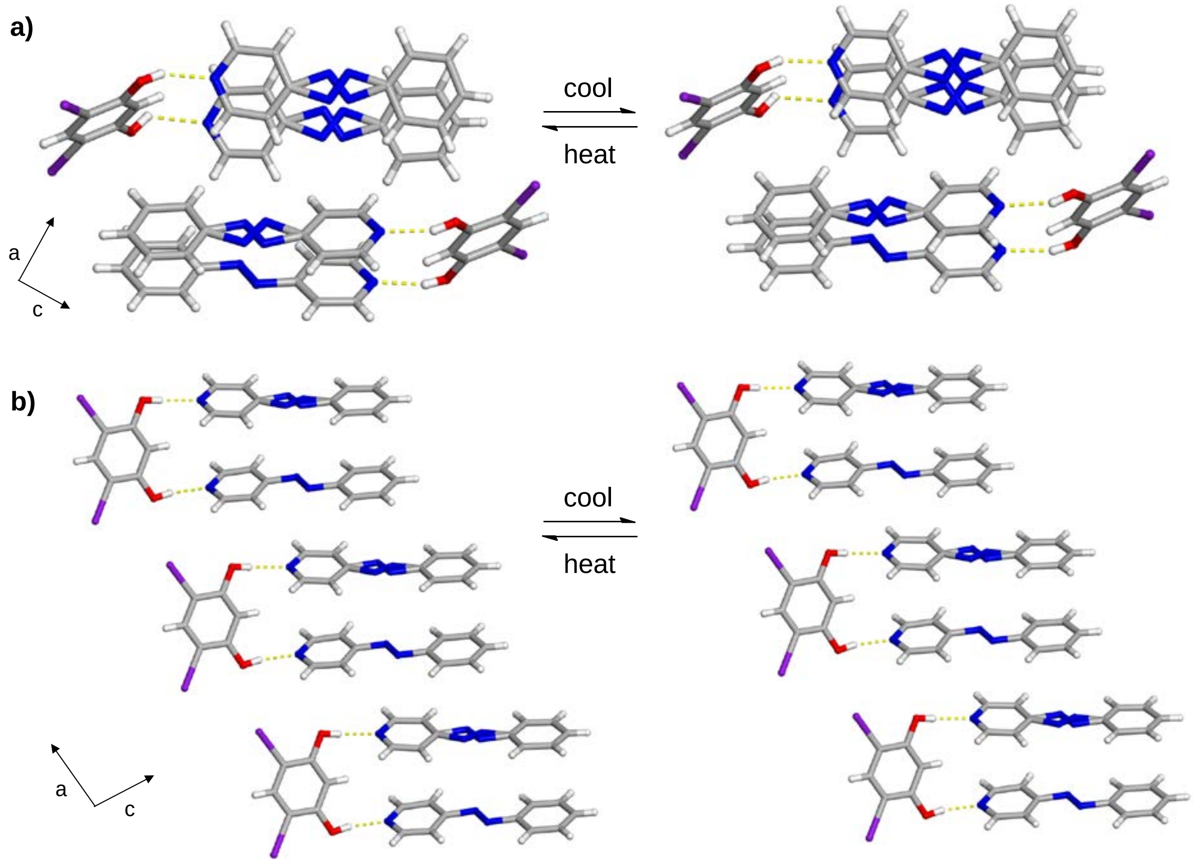

Fig. S4. Crystal structures of **3** at 290 and 190 K: (a) asymmetric unit containing three disordered azo groups and (b) extended parallel stacked column of one type of three-component assembly.

5) Differential Scanning Calorimetry

Differential scanning calorimetry was performed on powdered samples using a TA DSC Q100. Approximately 5 mg of sample was sealed in a crimped aluminum pan. The experiment was conducted using nitrogen purge. Samples were cooled from RT to 215 K, equilibrated, and warmed to RT. The cycle was repeated a second time unless otherwise noted.

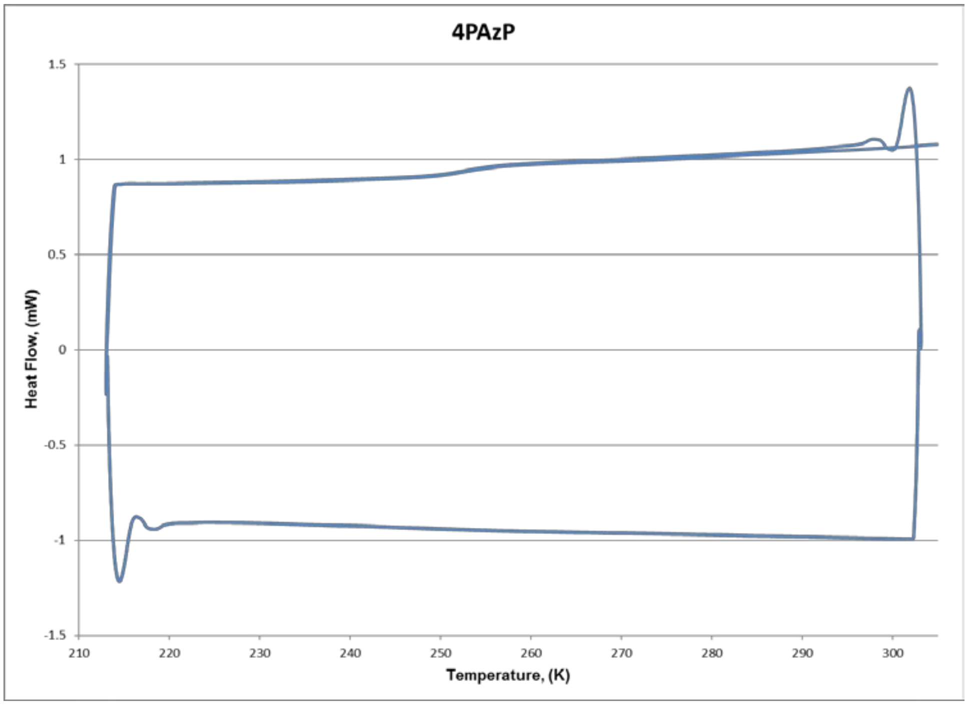

Fig. S5. DSC of **4PAzP** showing no phase transition. The small peaks at 30 and -60 °C are artifacts of the cooling cycle process.

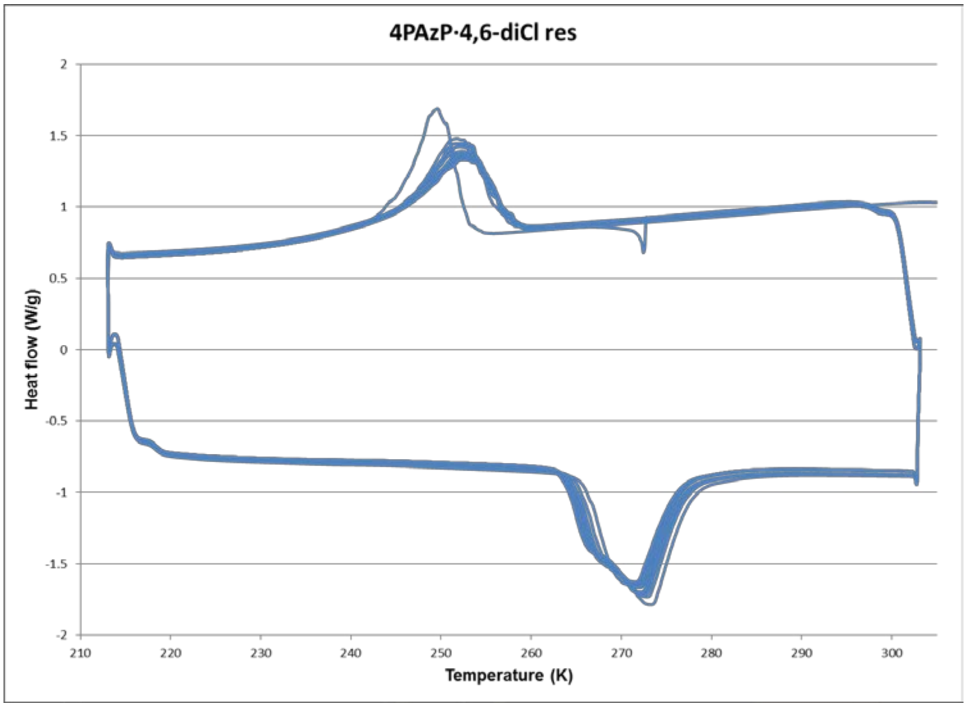

Fig. S6. DSC of **1** showing reversible phase transition. The cycle was repeated 15 times. See references 4-6 for hysteresis and peak shifts in a first-order phase transition.

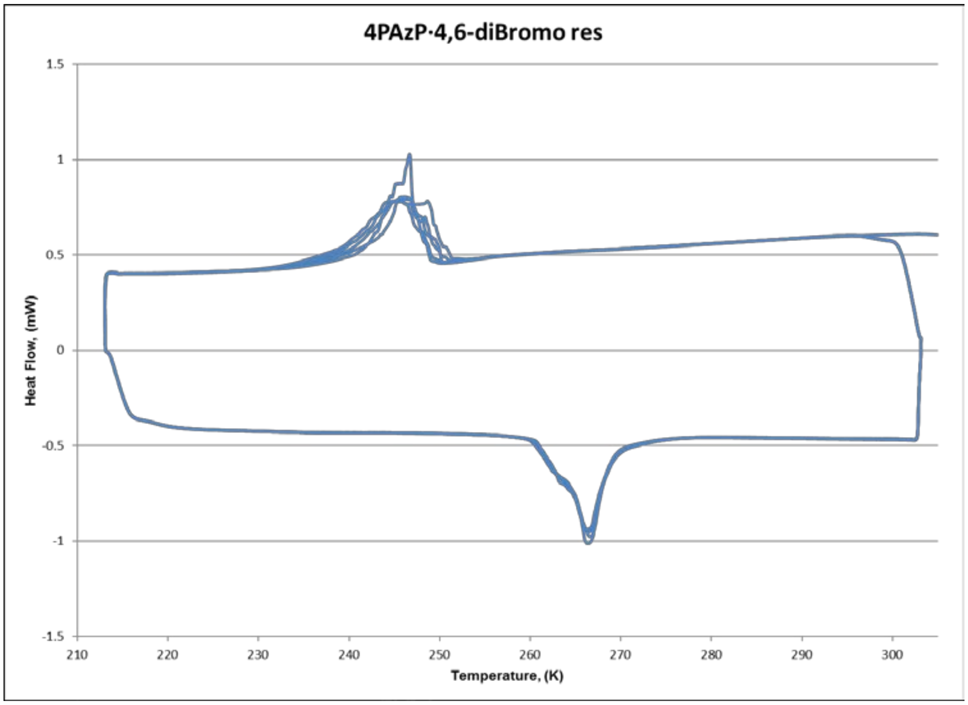

Fig. S7. DSC of **2** showing reversible phase transition. The cycle was repeated 5 times. See references 4-6 for hysteresis and peak shifts in a first-order phase transition.

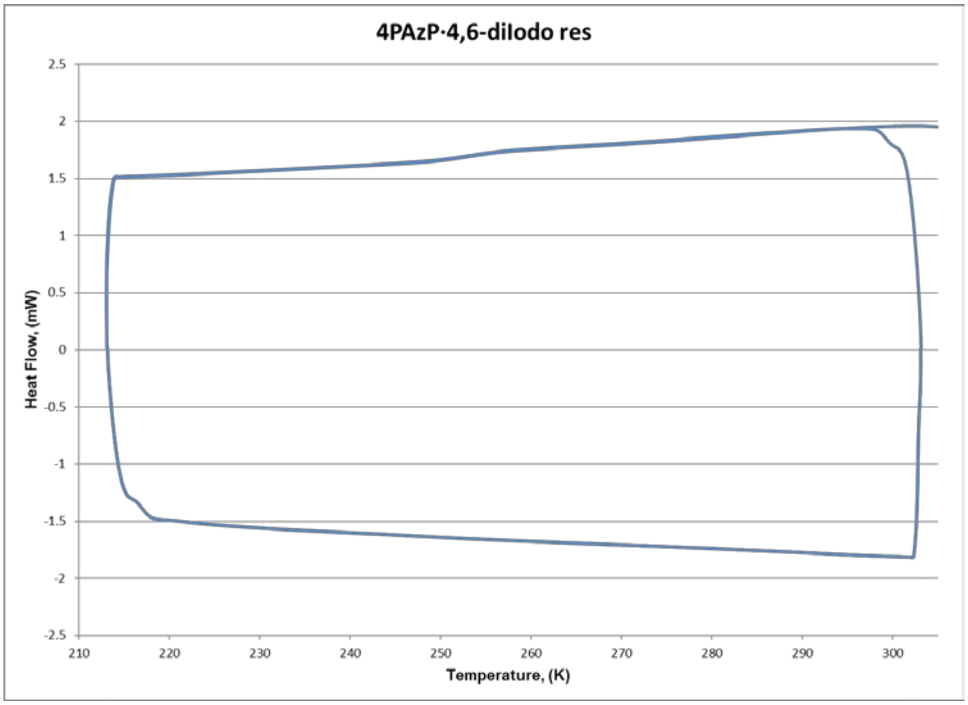

Fig. S8. DSC of **3** showing no phase transition.

6) Thermal expansion coefficients

The thermal expansion coefficients were calculated using the PAScal program.[7] The data from the unit cell determinations were used for **1** and **2**, and the crystallographic data sets were used for **3** and **4PAzP** (Tables S2-S4).

Table S6. Thermal expansion coefficients for co-crystals **1-3** and **4PAzP**.

Temperature range (K)	α_{x1} (MK^{-1})	α_{x2} (MK^{-1})	α_{x3} (MK^{-1})	α_{v} (MK^{-1})
**4PAzP·4,6-diCl res**				
290-260	-116	29	316	229
250-170	0.5	28	174	203
**4PAzP·4,6-diBr res**				
290-260	-51	58	193	201
250-170	10	111	125	249
**4PAzP·4,6-diIodo res**				
290-190	-13	74	135	197
**4PAzP**				
290-170	5	26	54	85

$\alpha_{x1,}$ $\alpha_{x2,}$ and α_{x3} are the thermal expansion coefficients for the principal crystallographic axes shown in Table S2.
α_{x1} is the volumetric thermal expansion coefficient.

Table S7. Approximate crystallographic axes for the calculated principal crystallographic axes.

Co-crystal	Approximate crystallographic axis for X1	Approximate crystallographic axis for X2	Approximate crystallographic axis for X3
**4PAzP·4,6-diCl res 290-260**	203	10-1	010
**4PAzP·4,6-diCl res 250-170**	203	-203	010
**4PAzP·4,6-diBr res 290-260**	100	010	001
**4PAzP·4,6-diBr res 250-170**	201	103	010
**4PAzP·4,6-diIodo res**	13-1	401	431
**4PAzP**	100	001	010

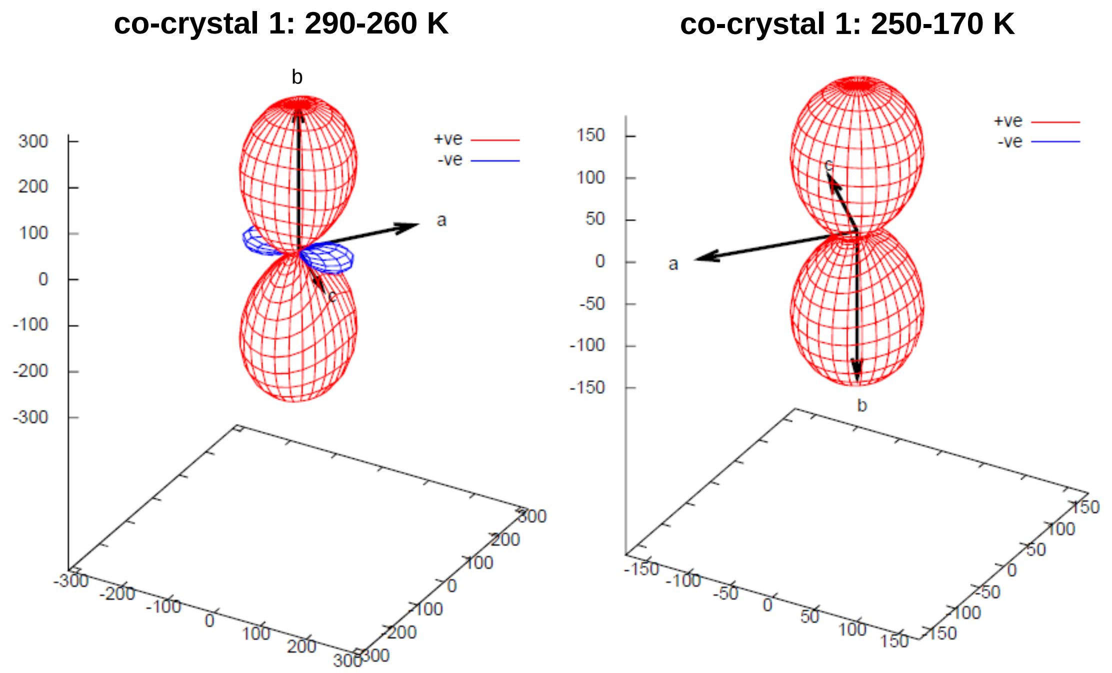

Fig. S9. Expansivity indicatrix representing the 3D plot of the calculated thermal expansion coefficients along the crystallographic axes for co-crystal **1**. Red represents PTE and blue represents NTE.

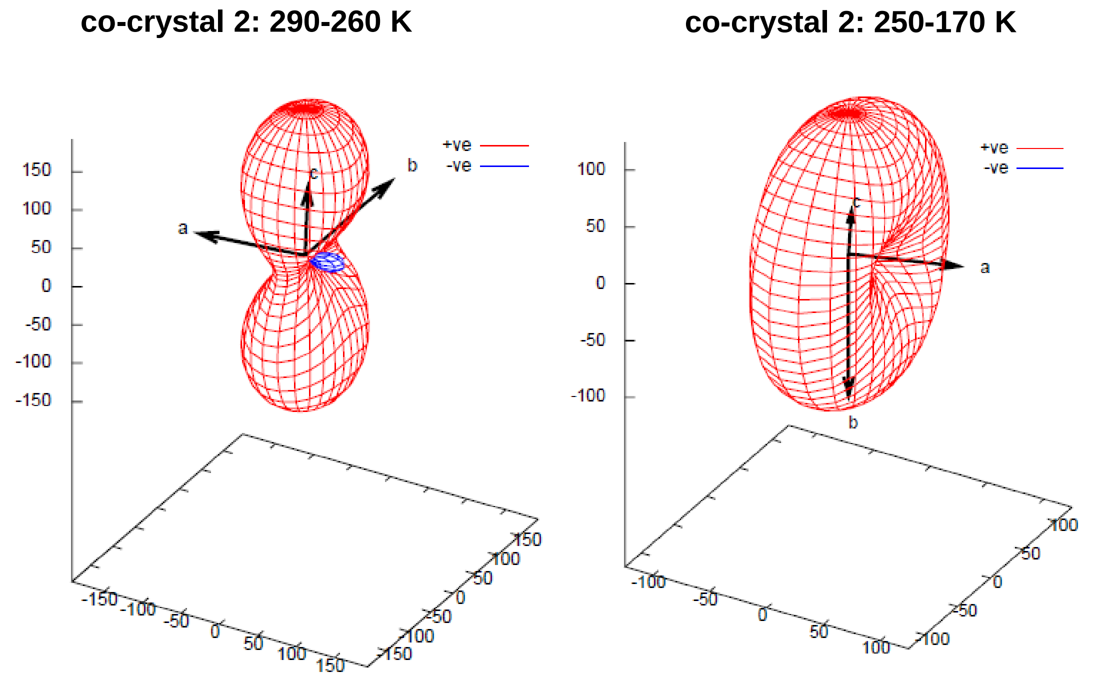

Fig. S10. Expansivity indicatrix representing the 3D plot of the calculated thermal expansion coefficients along the crystallographic axes for co-crystal **2**. Red represents PTE and blue represents NTE.

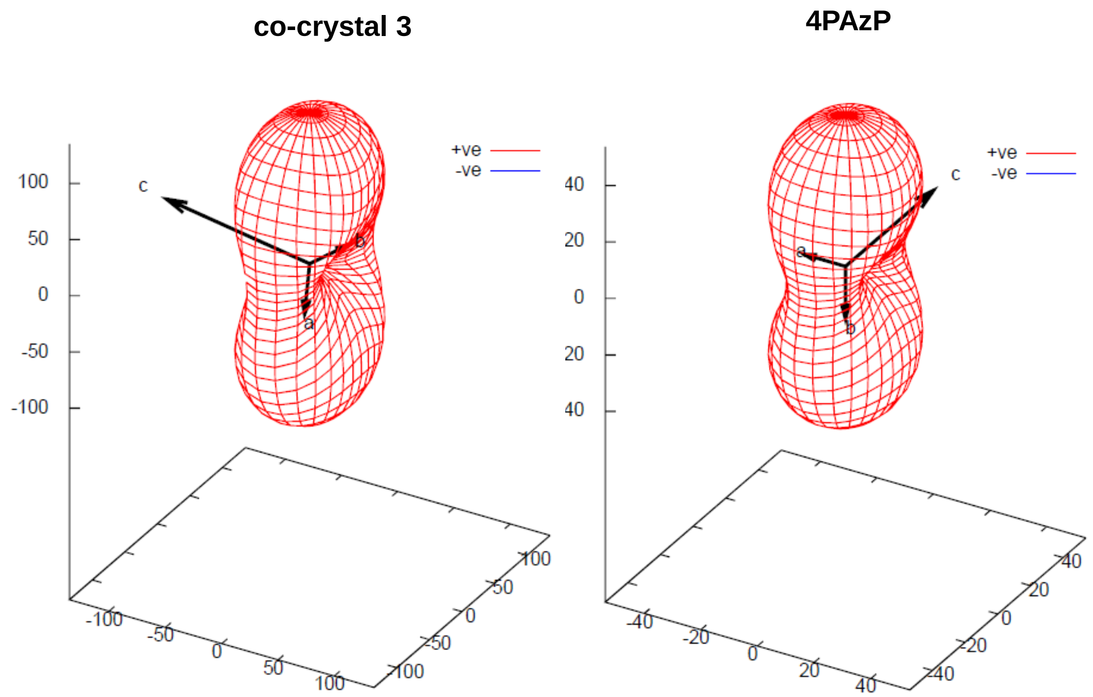

Fig. S11. Expansivity indicatrix representing the 3D plot of the calculated thermal expansion coefficients along the crystallographic axes for co-crystal **3** and **4PAzP**. Red represents PTE and blue represents NTE.

References

1. A. Bannwarth, *et al*., *Eur. J. Inorg. Chem*., 2012, **2012**, 2776.
2. G. M. A. Sheldrick, *Acta Crystallogr. Sect. A: Found. Crystallogr.*, 2008, **64**, 112.
3. O. V. Dolomanov, L. J. Bourhis, R. J. Gildea, J. A. K. Howard, H. Puschmann, *J. Appl. Cryst.*, 2009, **42**, 339.
4. R. Shang, G. C. Xu, Z. M. Wang, S. Gao, *Chem. Eur. J.* 2014, **20**, 1146.
5. V. Korthuis, N. Khosrovani, A. W. Sleight, *Chem. Mater*. 1995, **7**, 412.
6. S. R. Saha, *et al*., *Phys. Rev. B* 2012, **85**, 024525/1.
7. M. J. Cliffe, A. L. Goodwin, *J. Appl. Crystallogr*. 2012, **45**, 1321.
